# Adult obese mice suffer from chronic secondary brain injury after mild TBI

**DOI:** 10.1186/s12974-016-0641-4

**Published:** 2016-06-30

**Authors:** Matthew Sherman, Ming-Mei Liu, Shari Birnbaum, Steven E. Wolf, Joseph P. Minei, Joshua W. Gatson

**Affiliations:** Department of Surgery, University of Texas Southwestern Medical Center, Dallas, TX USA; Department of Neurological Surgery, University of Texas Southwestern Medical Center, 5323 Harry Hines Blvd., Dallas, TX 75390-9160 USA; Department of Psychiatry, University of Texas Southwestern Medical Center, Dallas, TX USA

**Keywords:** TBI, Inflammation, Obesity

## Abstract

**Background:**

A traumatic brain injury (TBI) event is a devastating injury to the brain that may result in heightened inflammation, neurodegeneration, and subsequent cognitive and mood deficits. TBI victims with co-morbidities such as heart disease, diabetes, or obesity may be more vulnerable to the secondary brain injury that follows the initial insult. Compared to lean individuals, obese subjects tend to have worse clinical outcomes and higher mortality rates after trauma.

**Methods:**

To elucidate whether obesity predisposes individuals to worse outcomes after TBI, we subjected adult lean and obese male/female mice to a mild TBI. The injury was administered using a controlled skull impact (CSI) device. Lean or obese 6-month-old C57 BL/6 mice were subjected once to a mild TBI. Additionally, at day 30 after injury, both the lean and obese mice were tested for increased anxiety using the open field test.

**Results:**

At day 30 after TBI, compared to the lean mice, we found heightened microglial (MG) activation in the cerebral cortex, corpus callosum, and hypothalamus. Another compelling finding was that, compared to the non-injured obese male control mice, the obese TBI mice had a decrease in the rate of weight gain and serum corticosterone levels at day 30 after injury. Additionally, the injured obese mice displayed higher levels of anxiety as determined by a significant decrease in time spent in the non-peripheral zones in the open field test. In contrast to the obese males, the obese female mice did not exhibit increases in the number of active MG in the brain, changes in weight gain/corticosterone levels, or increased anxiety at day 30 after TBI.

**Conclusions:**

The data presented here suggests that obese mice have worse outcomes compared to lean mice after mild TBI. Also, the obese males have worse outcomes than the injured female mice. This data may explain the sequela of chronic secondary brain injury in obese adults after a single mild TBI. Also, this report may help shape how the overweight/obese populations are monitored over the days and months following a TBI.

## Background

Each year in the USA alone, a third of a million persons are hospitalized for a traumatic brain injury (TBI) event, of which approximately 50,000 die. The devastating cost of TBI each year is approximately $77 billion in the USA. Despite advances in neurosurgical interventions and intensive care monitoring, many of the survivors of TBI do not fully recover and are left with permanent disability [1–4]. Mild TBI is the predominant form that is reported (~75 %) [5, 6] and is usually poorly managed, resulting in cognitive deficits later in life.

Previously, it has been determined that obese individuals produce higher levels of pro-inflammatory cytokines such as tumor necrosis factor-α, IL-1β, and IL-6, to name a few [7–9]. In the brain, links between obesity, neuro-inflammation, neurodegeneration, deficits in neurogenesis, and cognitive/mood disorders have been reported [10–25]. Previously, obese patients that experienced a blunt trauma were found to have more clinical complications and higher mortality rates than lean patients with similar injuries [26]. Another study found that college athletes with a high body mass index had a decrease in cognitive performance [27, 28]. Cognitive decline has also been observed in obese people compared to more lean individuals [29–32]. To date, there is no published data that defines the relationship between obesity, secondary brain injury, and neurological outcomes in TBI survivors. Data collected from both animals and humans has demonstrated that adipose tissue in obese individuals secretes a larger amount of pro-inflammatory factors compared to adipose tissue from more lean individuals. This increase in circulating inflammatory factors is detectable and is lowered once the adipose load is reduced. A high adipose tissue load for a significant amount of time is considered a chronic phenomenon [16]; thus, individuals in this state may be more vulnerable to secondary insults. Within their central nervous system (CNS), obese individuals experience a heightened state of inflammation. For example, in previous studies it was determined that an increase in inflammation in the CNS is involved in obesity-induced insulin resistance. Also, in a number of studies, mice that were fed a high-fat diet were found to have higher levels of activated microglia (MG) within brain regions such as the hypothalamus [33–35].

In the present study, we sought to characterize chronic secondary brain injury in lean and obese mice after mild TBI. We found that brain-injured adult obese male mice at a chronic post-injury time-point (30 days) have an increase in microglia activity throughout the brain, altered metabolism, and increased anxiety.

## Methods

### Animals

The experimental animals were housed and cared for by the Animal Resource Center (ARC) at University of Texas Southwestern Medical Center (UTSWMC), which is certified by the Association for Assessment and Accreditation of Laboratory Animal Care. All procedures listed in this article were approved by the UTSWMC Institutional Animal Care and Use Committee.

### Adult mouse obesity model

To establish the adult mouse obesity model, 2-month-old male and female C57 BL/6 mice were fed a high-fat diet (60 % kcal high-fat diet) for 4 months. The lean mice were fed a normal chow (10 % kcal normal diet) for the duration of the study. Prior to and after injury, the normal diet and obese animals were weighed. Mice were deemed to be obese once these mice experienced an approximate 25–30 % increase in body weight compared to the age-matched lean animals.

### Mouse TBI model and treatment

The Benchmark Stereotaxic Impactor (Leica Microsystems) was used to administer a mild TBI in the mice (~10 animals per group). In brief, adult (6-month-old) lean and obese C57 BL/6 mice were anesthetized using isoflurane (3 %) and placed in an adapted nosecone device. To maintain the animals’ body temperature during the TBI procedure, a heating pad was placed under the animals. The animals’ body temperature was monitored using a rectal thermometer. In this closed-skull TBI model, an incision was made to expose the skull and the impactor tip (4-mm flat tip) was aligned on the sagittal suture midway between the bregma (A/P −0.8 mm of the bregma) and lambda sutures. A single impact was delivered on the skull using the pneumatic cylinder at a velocity of 3 m/sec to a depth of 1.25 mm with a dwell time of 100 ms. The skin was closed with surgical wound clips. In all of the groups, at 5 min and 12 h after injury, the animals were treated with buprenorphine (0.05 mg/kg) to manage pain. At the indicated time-point, the animals were anesthetized and intra-cardially perfused with 0.9 % saline followed by 10 % phosphate-buffered formalin. The whole brain was removed, sliced, and stained for activated MG.

### Enzyme-linked immunosorbent assay

At the time of euthanasia, blood was collected from the animals, processed, and stored at −80 °C. In brief, prior to sacrificing the animal, blood was collected from the left ventricle, centrifuged, and stored at −80 °C until processed. The enzyme-linked immunosorbent assay (ELISA) method was used to detect serum levels of corticosterone (Abcam) in both the lean and obese control/TBI animals. To do so, 25 μl of each sample was loaded into each well. Each sample was tested in duplicate. The plate was incubated with the biotinylated corticosterone for approximately 2 h at room temperature. After the 2-h treatment, the plates were washed and subsequently treated with 50 μl of the conjugate and then chromogen for 30 min at room temperature. After the treatment with the chromogen, 50 μl of stop solution was added. The absorbance was detected at 450 nm. The values were compared to the standard curve, and the concentrations were determined.

### Immunohistochemistry

Following the fixation steps, paraffin sections were cut at 5 μm and fixed to the microscope slides. The sections were treated with hydrogen peroxide to inactivate endogenous peroxidase. The primary antibody, Iba1 (1:500 dilution; Wako Chemicals, Richmond, Virginia), was added to the sections, and the sections were treated overnight at 4 °C. Single-antigen detection was performed using the Alexa Fluor 647 donkey anti-rabbit secondary antibody (Abcam). The number of microglia was counted per field and characterized as being resting (ramified) or activated (hypertrophic or bushy). The stained slides were viewed using a Zeiss Imager A.2 microscope. The averages of nine different fields of view were calculated for each animal (counts from three fields of view/coronal slice (three total slices per animal)). A bar graph depicts the average number of positively stained cells for each group.

### Open field test

The control and mild TBI lean/obese animals were tested for anxiety and deficits in locomotor activity using the open field test. In brief, the mice were placed in the periphery of a novel open field environment (44 cm × 44 cm, walls 30 cm high) in a dimly lit room and allowed to explore for 5 min. The animals were monitored from above by a video camera connected to a computer that was running video-tracking software (EthoVision 3.0, Noldus, Leesburg, Virginia) to determine the time, distance moved, and number of entries into two areas: the periphery (5 cm from the walls) and the center (14 cm × 14 cm).

### Statistics

Data was obtained from no fewer than ten independent experiments using the Student’s *t* test unpaired or paired analysis. Groups were considered to be significantly different if *P* values were < .05 (SPSS; Chicago, Illinois). The data is presented as a bar graph/scatter plot depicting the mean + SD using GraphPad Software (San Diego, California). Groups were considered significantly different if *P* < .05.

## Results

### Obese male mice exhibited a reduction in the rate of weight gain and a significant decrease in blood corticosterone levels at day 30 after TBI

**Fig. 1:**
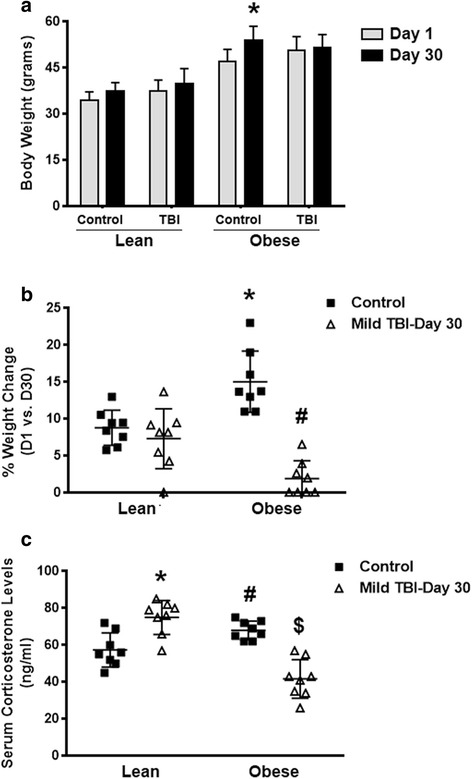
Brain-injured obese male mice exhibit decreases in the rate of weight gain and blood corticosterone levels. Body weight (**a**), % body weight change (**b**), and serum corticosterone levels (**c**) were measured in both lean/obese control and TBI animals (*n* = 10). Data was collected at days 1 and 30 after injury. **a** Paired Student’s *t* test: *P < .004 vs. obese control at day 1. **b** Unpaired Student’s *t* test: *P < .003 vs. lean control; ^#^ *P* < .0001 vs. obese control. **c** Unpaired Student’s *t* test: *P < .002 vs. lean control; ^#^ *P* < .01 vs. lean control; ^$^ *P* < .0001 vs. obese control

Body weight measurements were performed on the lean and obese mice at the time of injury (day 1) and at day 30 after TBI. Between days 1 and 30 after TBI, a slight increase (not significant) in body weight was observed in the lean control and TBI groups. In contrast, a significant increase (*P* < .004) in body weight was recorded for the non-injured obese control mice at day 30. No significant increase (*P* < .24) in body weight was observed at day 30 after TBI in the obese TBI group (Fig. 1a). Within the male lean control and lean TBI groups, the rate of weight gain (~8 % increase) within the 30-day test period was similar. The obese male control mice had an average increase in weight of 15 % within the 30-day period, which was significantly (*P* < .003) different than that of the control lean group. In contrast, the male obese TBI animals had a significant (*P* < .0001) decrease in the percentage of weight gain compared to the obese male control mice. These mice only had an average increase of weight of about 2 % over the 30-day testing period (Fig. 1b).

Additionally, in comparison to the obese male control mice, the obese mice that experienced a TBI had about a 43 % decrease (*P* < .0001) in the serum levels of corticosterone. In contrast, the lean TBI animals had an approximate 26 % increase (*P* < .002) in the serum levels of corticosterone, which was different from the age-matched controls. Also, the obese male control mice had a higher level (*P* < .01) of serum corticosterone compared to the lean male control mice (Fig. 1c).

### Obese female mice exhibited normal rates of weight gain at day 30 after mild TBI

**Fig. 2:**
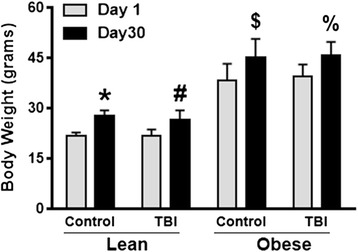
The rate of weight gain in brain-injured obese females is similar to non-injured controls. Body weight was measured in both lean/obese control and TBI female mice (*n* = 10). Data was collected at days 1 and 30 after injury. A paired Student’s *t* test was performed to determine differences in weight for the female mice within each group. *P < .008 vs. lean control; ^#^ *P* < .0007 vs. obese control; ^$^ *P* < .001 vs. obese control; ^%^ *P* < .0001 vs. obese control

To elucidate whether the female mice experience altered weight gain rates, we subjected lean and obese 6-month-old female mice to a mild TBI. At day 30 after TBI, we found that both control (*P* < .008) and TBI (*P* < .0007) lean females had significant increases within a 30-day period. Also, after TBI, the obese female control animals also experienced a significant increase (*P* < .001) in weight gain between days 1 and 30. In contrast to the obese male TBI animals, the injured obese female mice had a significant increase (~9 %; *P* < .0001) in weight gain compared to the obese control animals during the 30 days after TBI (Fig. 2). This finding is similar to the male (Fig. 1a) and female lean TBI group (Fig. 2). With respect to corticosterone levels, within the obese female group, no differences between the control and TBI animals were observed (data not shown).

### In contrast to females, the obese male mice had elevated MG activity in the brain at day 30 after TBI

**Fig. 3:**
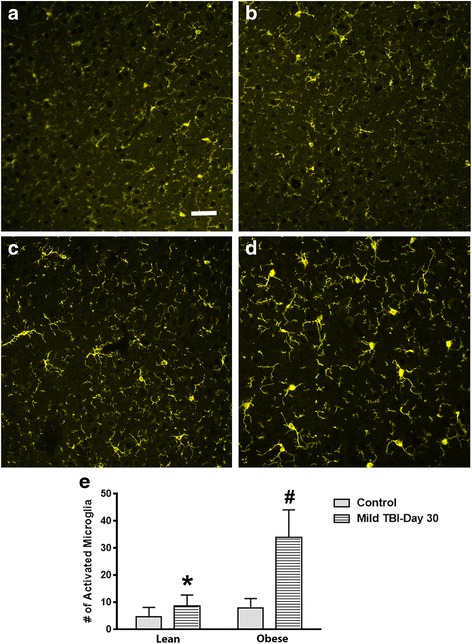
MG activity is increased at day 30 after TBI in the cerebral cortex of obese male mice after TBI. Compared to lean control (**a**), lean + TBI (**b**), and the obese control (**c**) groups, the obese mice subjected to a TBI exhibited more activated MG (**d**). *Scale bar* = **a**–**d** 100 μm. The increase in the number of activated MG was significant. **e** Unpaired Student’s *t* test: *P < .0001 vs. obese TBI mice; ^#^ *P* < .0001 vs. obese control mice. Approximately 10 animals per group were tested in this study

**Fig. 4:**
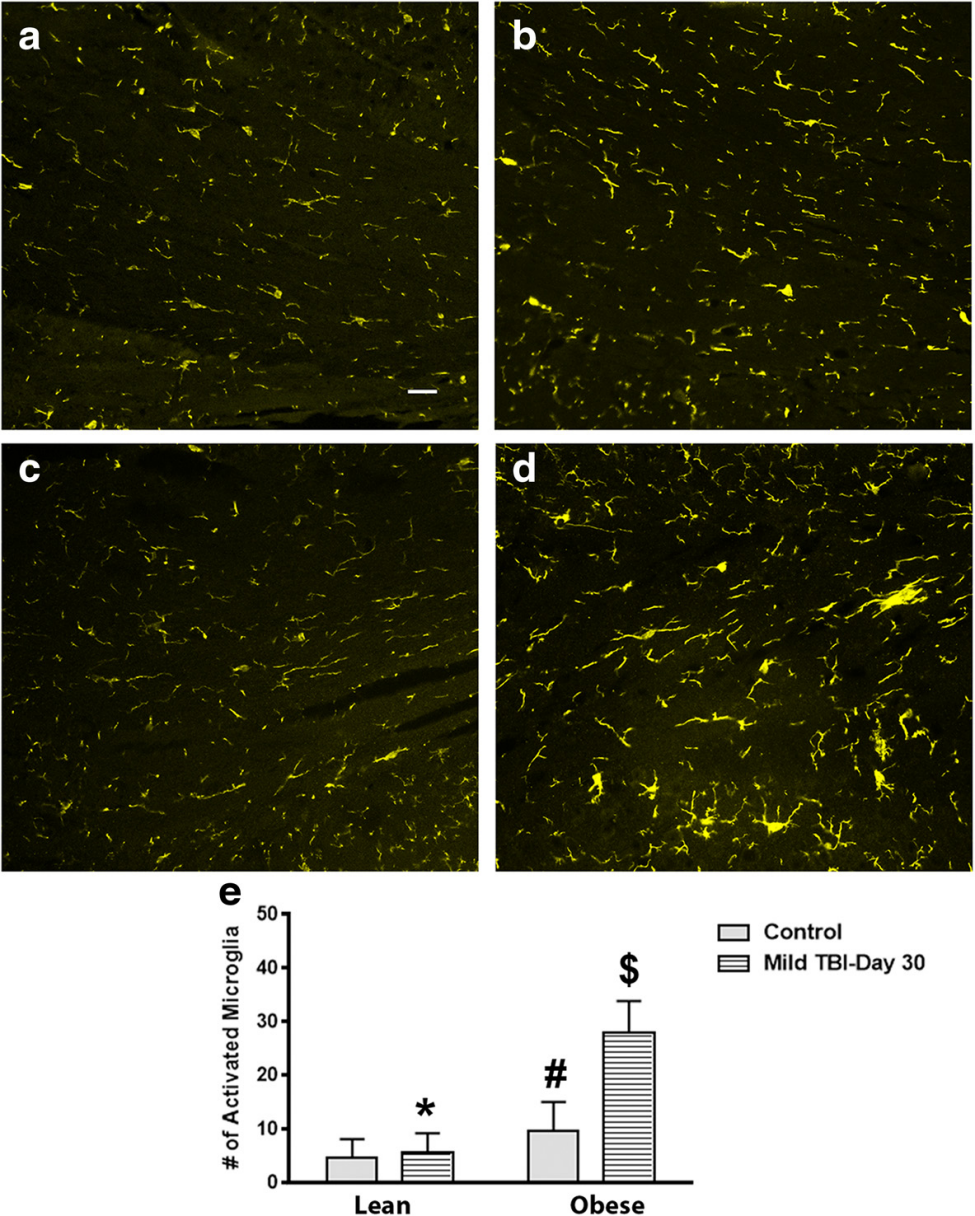
Obese male mice exhibit an increase in MG activation in the corpus callosum at day 30 after brain injury. MG activity in the corpus callosum of the obese male mice was increased (**d**) compared to the lean control (**a**), lean + TBI (**b**), and obese control (**c**) groups. *Scale bar* = **a**–**d** 100 μm. The bar graph presented in **e** illustrates significant differences between the reported groups. **e** Unpaired Student’s *t* test: *P < .001 vs. obese TBI mice; ^#^ *P* < .001 vs. lean control mice; ^$^ *P* < .001 vs. obese control mice. Approximately 10 animals per group were tested in this study

**Fig. 5:**
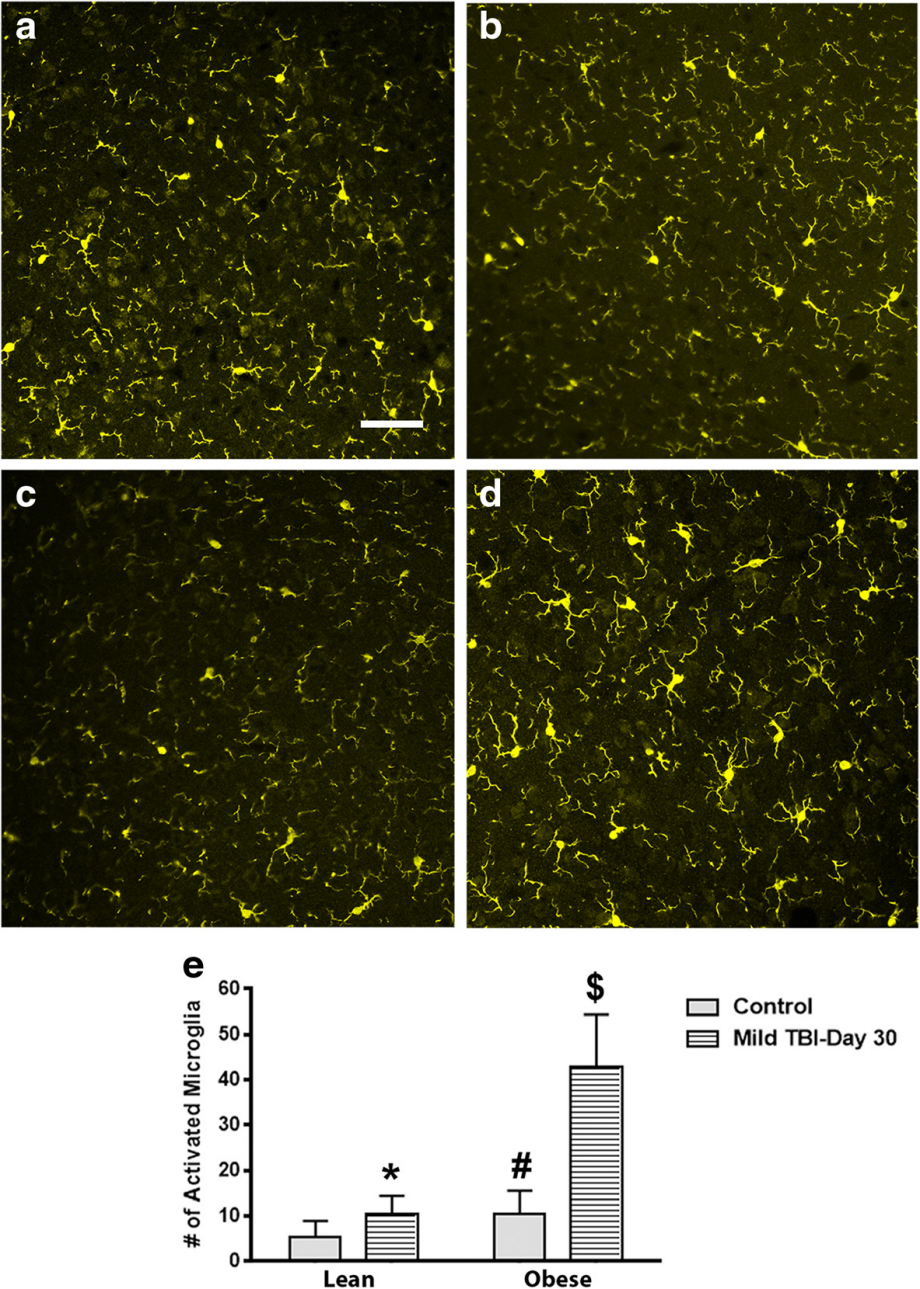
MG levels are significantly increased in the hypothalamus of the obese male TBI mice at day 30 after injury. Compared to lean control (**a**), lean + TBI (**b**), and the obese control (**c**) groups, the obese TBI mice had a significantly higher level of activated MG within the hypothalamus (**d**). *Scale bar* = **a**–**d** 100 μm. The bar graph presented in **e** illustrates significant differences between the reported groups. **e** Unpaired Student’s *t* test: *P < .0001 vs. obese TBI mice; ^#^ *P* < .03 vs. lean control mice; ^$^ *P* < .0001 vs. obese control mice. Approximately 10 animals per group were tested in this study

**Fig. 6:**
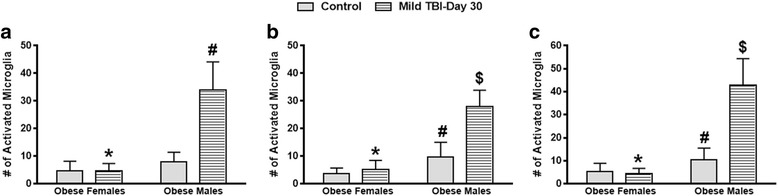
In contrast to obese male TBI mice, obese female mice exhibit significantly lower levels of active MG in the brain at day 30 after TBI. Significant differences between males and females with respect to MG activity were observed at day 30 after TBI in the parietal cortex (**a**), corpus callosum (**b**), and hypothalamus (**c**). **a** Unpaired Student’s *t* test: *P < .0001 vs. obese TBI males; ^#^ *P* < .0001 vs. obese control males. **b** Unpaired Student’s *t* test: *P < .0001 vs. obese TBI males; ^#^ *P* < .009 vs. obese control females; ^$^ *P* < .001 vs. obese control males. **c** Unpaired Student’s *t* test: *P < .0001 vs. obese TBI males; ^#^ *P* < .03 vs. obese control females; ^$^ *P* < .0001 vs. obese control males. Approximately 10 animals per group were tested in this study

Following TBI, the brains of lean and obese 6-month-old mice were isolated, fixed, and stained for activated microglia at day 30 after injury. Compared to non-injured controls, the brain-injured lean mice had a similar ratio of ramified to hypertrophic/bushy microglia within the parietal cortex, corpus callosum, and hypothalamus. To assess whether the obese mice exhibit higher levels of activated microglia within these brain regions, the obese mice were subjected to a mild-to-moderate TBI. Interestingly, compared to the injured lean mice, a significant increase of activated microglia was detected in the parietal cortex (*P* < .0001), corpus callosum (*P* < .001), and hypothalamus of the obese male TBI mice (*P* < .0001; Figs. 3, 4, and 5). In the parietal cortex, the heightened MG response was found to border the injury zone. Minor cortical lesions were also observed. This pattern of MG activity was not observed in the lean/obese TBI or control animals. A fourfold increase (*P* < .0001) was observed between the obese male control and obese male TBI mice (Fig. 3). In contrast to the parietal cortex, a more diffuse pattern of MG activation was observed in the corpus callosum and hypothalamic brain regions (Figs. 4 and 5). Heightened MG activation was concentrated in the splenium region of the corpus callosum located directly below the injury site. In comparison to the obese male control mice, the obese TBI animals had an approximate threefold increase (*P* < .001) at day 30 after mild TBI. Another interesting finding is that MG activity in the obese male control mice was significantly (*P* < .001) higher than that in the lean male control animals, suggesting that a chronic obese state increases MG activity in the corpus callosum (Fig. 4). In the hypothalamus of the injured obese male mice, compared to the obese male controls, MG activation was also increased (*P* < .0001) primarily in the ventromedial nucleus (VMN) of the hypothalamus. The pre-injury/baseline MG activity levels of the obese male control mice were also significantly (*P* < .03) higher than those of the lean male control animals (Fig. 5). In contrast to the male mice, the control and TBI obese female mice had similar numbers of activated MG when compared to the lean control and TBI groups. The MG activity in the obese female TBI mice was significantly lower than the MG activity in the obese male TBI mice within the parietal cortex (*P* < .0001), corpus callosum (*P* < .0001), and hypothalamic brain regions (*P* < .0001; Fig. 6).

### Compared to lean mice, the obese male mice experienced heightened anxiety after TBI

**Fig. 7:**
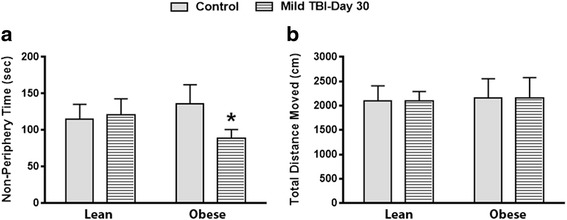
Obese male TBI mice display increased anxiety at day 30 after TBI. At day 30 after TBI, a significant (*P < .008) decrease in the time spent in the non-peripheral zone was observed in obese male mice (**a**). No difference in overall activity was observed since no difference in the total distance moved was observed between all groups (**b**). Approximately 10 animals per group were tested in this study

To elucidate whether increased inflammation in the brain results in altered exploratory behavior and anxiety levels, the non-injured and injured lean and obese male/female mice were subjected to the open field test at day 30 after TBI. The non-injured lean male control and TBI mice exhibited similar behaviors. These animals spent a similar amount of time in the periphery/non-periphery areas of the field. In contrast, the obese male TBI mice displayed thigmotaxis (increased anxiety) since the mice spent significantly less (*P* < .008) time in the non-periphery zones. The non-injured obese control mice spent a similar amount of time in the periphery/non-periphery zones as the lean animals (Fig. 7a). Locomotor activity was normal for all groups tested since we observed no differences in the total distance traveled by the lean and obese mice (Fig. 7b). No deficits were observed in the lean and obese female groups.

## Discussion

After TBI, overweight/obese individuals experience worse outcomes when compared to more lean people with similar diagnoses. The exact mechanisms to describe these differences are unknown. In this study, we found that adult obese male mice that suffered a mild brain injury experienced altered physiological responses compared to lean and obese female mice. More specifically, the brain-injured obese male mice exhibited higher levels of anxiety, significantly reduced corticosterone levels, and had a reduced rate of weight gain over a 30-day period (Figs. 1, 2, and 7). Also, the obese male TBI mice had a significant increase in inflammation (MG activity) in the brain. To our surprise, multiple brain regions such as the cerebral cortex, corpus callosum, and hypothalamus were affected (Figs. 3, 4, and 5). As expected, the baseline MG activity levels were found to be slightly elevated in the non-injured obese control mice compared to the lean animals and trauma to the head region of the obese mice exacerbated the pro-inflammatory state of the obese mouse brain.

Here, we sought to characterize obesity as a risk factor for developing chronic secondary brain injury after TBI. In this model, we hypothesize that after TBI, observations of decreased blood corticosterone levels and weight gain rates in the obese male mice are a result of persistent inflammation in brain regions such as the hypothalamus. This chronic inflammatory state in the brain is thought to result in a disruption of the hypothalamic pituitary adrenal axis (HPA axis) and subsequent behavioral deficits as observed in Fig. 7. We also hypothesize that the decrease in the rate of weight gain (obese males only) that was observed in this study is a result of hyper-MG activity in the hypothalamus, an altered HPA axis, and a reduction in plasma corticosterone levels.

The HPA axis is a complex interaction between the hypothalamus, pituitary gland, and the adrenal glands. These interactions are key for the control of a neuroendocrine system that regulates energy storage/expenditure, stress, and mood, to name a few [36]. In a number of studies, it was determined that obese individuals may have a hyper-responsive or dysregulated HPA system [37, 38]. As demonstrated here, heightened inflammation in the hypothalamus of the obese mice was increased and appeared to result in a reduction of corticosterone in the blood. Previous studies have demonstrated that altered HPA activity and corticosterone levels may result in a reduction of weight over time [39]. Also, damage to various regions of the hypothalamus such as the VMN (region that regulates body weight) results in reduced weight gain [40–42]. A major difference observed in this study was that the lean TBI animals had a significant increase in blood corticosterone levels, whereas the obese male TBI animals had a significant decrease in blood corticosterone levels. This difference in blood corticosterone levels at day 30 after TBI may explain why the rate of weight gain was significantly different between the lean and obese male TBI animals.

Another important finding is that other brain regions such as the cerebral cortex and corpus callosum exhibited significant increases in the number of activated MG in the obese male TBI mice. In addition to injury to the hypothalamus, damage to the cerebral cortex and corpus callosum may exacerbate the level of secondary brain injury and result in a heightened state of anxiety or the development of other cognitive deficits and/or neurodegeneration in the obese TBI mice. In addition to the findings reported here, other studies have also defined an association between high-fat diets and a plethora of neurological disorders (anxiety, manic depressive disorder, etc.) and neurodegenerative diseases (Parkinson’s disease, Alzheimer’s disease) [18–21, 43]. These published reports conclude that mice on high-fat diets may be more susceptible to a secondary brain injury that persists for days, weeks, and even months after injuries such as TBI.

Another interesting finding is that the obese female TBI mice have a significantly lower level of MG activity in the parietal cortex, corpus callosum, and hypothalamus compared to the obese male TBI mice. More importantly, the MG activity levels in the injured obese female mice were found to be similar to those of the lean TBI group (Figs. 3, 4, 5, and 6). Numerous animal and human studies in the TBI field have characterized outcomes in males and females with respect to secondary brain injury after TBI. To date, data is lacking that demonstrates that obese females experience better outcomes after TBI than obese males [44–47]. In this study, we hypothesized that obese males have worse outcomes and we propose that obesity is a risk factor for chronic secondary brain injury and neurodegeneration in obese males after TBI. Previously, multiple factors such as fat distribution, steroid hormones, and age have been found to contribute to differences observed with outcomes between males and females [48–50], which may explain why the obese females experienced better outcomes than males in this study. Further investigations are warranted to elucidate the mechanisms that render obese males more vulnerable to the initial trauma and chronic secondary brain injury.

## Conclusions

In conclusion, data is lacking that defines which individuals are the most susceptible to the detrimental effects of secondary brain injury. By increasing our knowledge of what factors and/or co-morbidities exacerbate neural injury after TBI, we can establish better monitoring/treatment paradigms to protect such vulnerable populations. Novel data presented here strongly suggests that obesity is a risk factor for chronic secondary brain injury in male mice.

## Abbreviations

ARC, Animal Resource Center; CNS, central nervous system; CSI, controlled skull impact; HPA, hypothalamic pituitary adrenal; MG, microglia; TBI, traumatic brain injury; UTSWMC, University of Texas Southwestern Medical Center; VMN, ventromedial nucleus
